# An Agent-Based Model of Cooperation and Competition in Organizational Systems

**DOI:** 10.3390/e28090965

**Published:** 2026-08-29

**Authors:** D. S. Fonte, L. H. A. Monteiro

**Affiliations:** 1 Escola de Engenharia, Universidade Presbiteriana Mackenzie, São Paulo 01302-907, SP, Brazil; danilo.fonte@mackenzista.com.br; 2 Escola Politécnica, Universidade de São Paulo, São Paulo 05508-010, SP, Brazil

**Keywords:** agent-based model, cooperation, Gini coefficient, organizational dynamics, prisoner’s dilemma, reputation, Shannon entropy

## Abstract

This study proposes a game-theoretic agent-based model to investigate cooperation and competition in workplace environments. The iterated prisoner’s dilemma is implemented on a two-dimensional lattice, where agents interact locally and accumulate wealth over time. Two types of agents are considered: fixed probabilistic cooperators, who adopt a constant cooperation probability, and adaptive probabilistic cooperators, whose behavior depends on their accumulated wealth and reputation. Population composition and wealth distribution are quantified using Shannon entropy and the Gini coefficient. Numerical simulations show that fixed cooperators tend to predominate and accumulate higher average wealth. The simulations also show that increasing tolerance to defections raises average wealth and reduces inequality. In contrast, a higher cooperation probability increases wealth but may also amplify inequality. These results highlight the role of reputation and performance targets in shaping cooperation in organizational environments.

## 1. Introduction

To cooperate is to act jointly with others toward a common objective, allocating resources that are not exclusively directed to immediate individual benefit. To compete is to act against others in order to achieve an individual objective, allocating resources primarily to maximize one’s own benefit at the expense of others. Cooperation and competition are central aspects of human life. A child survives and reaches adolescence through the sustained cooperation of others, most often parents or caregivers; however, siblings may compete for their attention. During the schooling phase, students usually cooperate with peers by sharing their understanding of classroom material; nevertheless, some compete for higher academic performance, in sports activities, or for preferred romantic partners. In adulthood, work is commonly carried out in collaborative environments, but competition for recognition remains, driven by the pursuit of better positions and higher salaries. This study proposes an agent-based model to investigate cooperation and competition among employees within organizations.

Even in collaborative settings, individual incentives may encourage actions that prioritize personal gain over collective outcomes. In workplace environments, competitive behavior may involve withholding information, claiming disproportionate credit for collective efforts, or adopting strategies that benefit the individual at the expense of group performance. These situations reflect a fundamental tension between cooperation and self-interest, which has been formally analyzed within the framework of game theory [1,2,3,4,5,6,7,8,9,10,11]. In game-theoretic terms, such self-interested behavior is described as defection, i.e., the choice to prioritize individual payoff over cooperative outcomes.

The emergence of cooperation among individuals seeking to maximize their own payoffs is one of the central themes in game theory, particularly in studies of the prisoner’s dilemma [1,2,3,4,5,6,7,8,9,10,11]. In this two-player game, each player receives a payoff *R* for mutual cooperation and a payoff *P* for mutual defection. If one defects while the other cooperates, the defector receives *T* and the cooperator receives *S*. By assumption, T>R>P>S and R>(T+S)/2 [1,2,3,4,5,6,7,8,9,10,11]. From an individual perspective, defection is therefore the rational choice, as it maximizes the payoff regardless of the other player’s action and avoids the lowest outcome *S*. However, if both players defect, each receives *P*, which is lower than *R*, the payoff associated with mutual cooperation. The dilemma arises because individually rational strategies lead to a collectively suboptimal outcome, highlighting the conflict between self-interest and mutual benefit.

In a single interaction, defection is the rational choice. However, cooperation can be sustained in the iterated prisoner’s dilemma, where players interact repeatedly over many time steps [1,5]. The prevailing strategy depends on the scenario under consideration; for instance, on the presence or absence of noise [1,5,12,13,14], understood as random perturbations that affect the perception of the opponent’s strategy or the implementation of one’s own strategy. These perturbations can significantly influence the resulting dynamics.

The emergence and persistence of cooperation in the iterated prisoner’s dilemma have been extensively studied in structured populations since the late twentieth century [15]. These studies include spatial lattices [16], heterogeneous coupling between interdependent lattices [17], lattices with negative links [18], random networks [19], small-world networks [20], scale-free networks [21], single-scale networks with dynamical linking [22], random networks with multiple interconnected layers [23], and multilayer networks consisting of coupled scale-free and small-world layers [24].

In addition, several mechanisms to promote cooperation have been introduced in the spatially structured, iterated prisoner’s dilemma, including player reputation [25], player mobility [26], success-driven increases in player influence [27], costly punishment of defectors [28], elimination of players with low accumulated payoffs [29], heterogeneity in the imitation of more successful neighbors [30], diversity in social influence and investment strategies [31], preferential imitation of high-reputation players [32], decisions based on own and counterpart payoffs in another lattice [33], strategy updating based on past strategies and memory length [34], trust profiles [35], players’ emotional states [36], heterogeneously stochastic interactions [37], a pre-play communication stage [38], sharing of neighbors’ payoff information [39], and rewiring based on payoff aspiration levels [40].

Other well-known games employed to investigate the evolution of cooperation include the snowdrift game [11,21,41] and the ultimatum game [42,43,44]. The model proposed here, however, is based on the classic prisoner’s dilemma, played iteratively on a two-dimensional lattice.

Organizations can be considered complex systems [45,46,47], and game theory has provided a formal framework for capturing the strategic behavior of individuals within them. For example, one study showed that cooperation in organizations can be modeled as an iterated prisoner’s dilemma, in which social relations among employees (such as shared community ties or informal relationships) serve as an additional enforcement mechanism, strengthening cooperation through trust and reputation effects [48]. A game-theoretic field experiment using the prisoner’s dilemma with workers in social cooperatives found that cooperative behavior in the workplace is influenced by organizational features (such as perceived fairness and the degree of social proximity among employees) and by individual dispositions (such as intrinsic motivation and prior job preferences) [49]. Another study based on the prisoner’s dilemma reported that the cooperative behavior of managers occupying central positions in workplace networks can enhance organizational performance and reduce employees’ stress levels [50].

Previous evolutionary-game models have considered reputation, aspiration, and agent elimination through different mechanisms. Reputation has been associated with punishment behavior [25] or used to favor the imitation of highly reputable players [32]. Aspiration levels have been employed to guide strategy updating and behavioral adaptation [30,51], and elimination mechanisms based on accumulated payoff have also been investigated [29]. In the present model, reputation decreases after defections, determines agent removal, and, together with accumulated wealth, affects the cooperation probability of adaptive agents.

Thus, the main contribution of this study is the joint consideration of these mechanisms in a spatially structured, iterated prisoner’s dilemma with two distinct behavioral types and an explicit replacement rule, interpreted in the context of workplace environments. The proposed framework can provide insights into how organizational policies influence cooperation, wealth distribution, and collective performance.

The remainder of this manuscript is structured as follows. In Section 2, a game-theoretic agent-based model for investigating cooperation and competition in the workplace is introduced. In Section 3, the metrics used to evaluate the observed dynamics are presented. In Section 4, the results obtained from numerical simulations are reported. In Section 5, these results are discussed from an organizational standpoint.

## 2. Model

Consider a two-dimensional lattice of size $\sqrt{N}\times \sqrt{N}$, such that each site of the lattice is occupied by one of the *N* agents forming the population. Each agent interacts with its four nearest neighbors, namely those to the north, south, east, and west. This connectivity pattern is known in the cellular automaton literature as a von Neumann neighborhood of radius one [52]. To ensure that all agents have the same number of neighbors, the top edge is connected to the bottom edge, and the left edge to the right edge. The use of these periodic boundary conditions eliminates boundary effects.

The regular lattice adopted here should not be interpreted in terms of physical proximity among employees in the workplace. Instead, it represents a simplified network of professional interactions, in which each employee interacts more frequently with a limited number of colleagues directly involved in their routine activities. In this framework, the four nearest neighbors correspond to the employee’s most relevant professional contacts. Although real organizational networks are more heterogeneous, workplace interactions are often structured around teams or departments, resulting in repeated interactions among relatively stable sets of employees. The regular lattice captures this predominance of local interactions without requiring additional assumptions about the underlying contact network. Furthermore, this connectivity pattern provides a baseline interaction framework commonly employed in studies of spatial evolutionary games [15,16,17,27,32,33,34,36,37,39,40].

In the prisoner’s dilemma, the actions available to each agent in each interaction are *C*, for cooperation, and *D*, for defection. The corresponding payoffs are *T* (if the agent chooses *D* and the opponent chooses *C*), *S* (if the agent chooses *C* and the opponent chooses *D*), *R* (if both choose *C*), and *P* (if both choose *D*), with T>R>P>S and R>(T+S)/2, as described in Section 1 [1,2,3,4,5,6,7,8,9,10,11]. From an organizational perspective, the temptation payoff *T* represents the individual benefit obtained by exploiting a cooperating counterpart, for instance, through disproportionate credit appropriation. The reward *R* corresponds to mutually beneficial and productive collaboration between employees. The punishment payoff *P* reflects low joint productivity resulting from mutual defection or lack of cooperation. Finally, the sucker’s payoff *S* captures situations in which a cooperating employee is exploited because their effort is neither properly recognized nor reciprocated.

In the model, the *i*-th agent (i=1,2,…,N) is characterized by three variables: $p_{i}\left(t\right)$, which is the probability of playing *C* at time step *t*, $w_{i}\left(t\right)$, which is the wealth accumulated by the agent up to time step *t*, and $r_{i}\left(t\right)$, which represents the agent’s reputation at that time step. At the initial time step, $w_{i}\left(0\right)=0$ and $r_{i}\left(0\right)=r_{0}$ for all agents, where $r_{0}$ is their initial reputation.

The concept of reputation in game theory was first introduced in analyses of the iterated prisoner’s dilemma [53]. Since then, reputation has been used in various ways; for instance, as a prestige metric that prompts agents to adopt the strategy of the most reputable neighbor [32], or as a marker of willingness to punish opponents [25]. By contrast, the model proposed here treats reputation as an internal variable, and not as a public signal to other agents.

At each time step, every agent plays the prisoner’s dilemma with its four neighbors. Thus, it probabilistically chooses to play *C* or *D*, and the payoffs from these four interactions are summed. The quantity $w_{i}\left(t\right)$ represents the cumulative rewards obtained over time. A simulation consists of τ time steps.

There are type-1 and type-2 agents. Type-1 agents cooperate with a constant probability $p_{i}\left(t\right)=q$ (and thus choose *D* with probability 1−q), independently of $w_{i}\left(t\right)$ and $r_{i}\left(t\right)$. Type-2 agents cooperate with probability $p_{i}\left(t\right)$ given by:

(1) $$p_{i}\left(t\right)=q\left[1-\left(\frac{w_{i}\left(t\right)}{g}\right){\left(\frac{r_{i}\left(t\right)}{r_{0}}\right)}^{2}\right]$$

where *g* is the agent’s cumulative wealth goal. This goal is related to the notion of an aspiration level, which has been used in previous studies of adaptive behavior and game-theoretic dynamics [40,51,54]. In Equation (1), q∈[0,1], $r_{i}\left(t\right)\in \left[0,r_{0}\right]$, and $p_{i}\left(t\right)=0$ for $w_{i}\left(t\right)\geq g$, as discussed below. The resulting values of $p_{i}\left(t\right)$ are thus restricted to the interval [0,1]. The multiplicative structure involving $\left(w_{i}\left(t\right)/g\right)$ and ${\left(r_{i}\left(t\right)/r_{0}\right)}^{2}$ in Equation (1) was adopted as a simple way to represent the joint influence of accumulated wealth and reputation on the cooperation probability of type-2 agents. Alternative formulations could involve a linear combination of these two factors, requiring additional weighting parameters and, consequently, additional modeling assumptions.

For both types of agents, the variable $r_{i}\left(t\right)$ is initialized at its maximum value, $r_{0}$, and decreases by one unit for each defection, i.e., each time the agent plays *D*. When the agent plays *C*, it remains unchanged. This variable represents the agent’s credibility, which, once diminished, is assumed not to recover within the time scale considered. When $r_{i}\left(t\right)=0$, the *i*-th agent is removed and is replaced at time t+1 by another agent, which starts with reputation $r_{0}$ and zero wealth. Thus, wealth is reset only for newly introduced agents, whereas remaining agents retain their accumulated wealth throughout the simulation. The replacement of a removed agent is random, with equal probability for each type. This replacement mechanism is more consistent with workplace environments, since the personality of a newly hired employee is typically not known in advance. Hence, in the absence of information favoring one behavioral profile over the other, newly recruited employees are assumed to have the same probability of belonging to either type.

The quadratic term ${\left(r_{i}\left(t\right)/r_{0}\right)}^{2}$ was introduced to make type-2 agents more sensitive to reputation loss. Since $0\leq (r_{i}\left(t\right)/r_{0})\leq 1$, squaring this ratio decreases its value. As this quantity is subtracted from 1 in Equation (1), the resulting cooperation probability becomes larger than it would be with a linear dependence. Consequently, the cooperation probability increases more rapidly as reputation declines. This choice reflects the assumption that agents experiencing reputation loss may increase their cooperative behavior in an attempt to avoid removal from the organization.

Note that at t=0; since $w_{i}\left(0\right)=0$, a type-2 agent chooses *C* with probability $p_{i}\left(0\right)=q$, and therefore initially behaves as a type-1 agent. As time progresses, however, its cooperation probability depends on both reputation and accumulated wealth. For a given value of $w_{i}\left(t\right)$, a lower reputation increases $p_{i}\left(t\right)$, whereas, for a given reputation $r_{i}\left(t\right)$, larger accumulated wealth decreases $p_{i}\left(t\right)$. When $w_{i}\left(t\right)\geq g$ for a type-2 agent, $p_{i}\left(t\right)$ is set to zero, and this agent becomes a persistent defector, i.e., it plays *D* exclusively as long as $r_{i}\left(t\right)>0$. This discontinuous transition was introduced as an idealized representation of situations in which the attainment of individual goals substantially reduces the incentives for cooperative behavior. Although smoother adjustments in cooperation probability could also be considered, the present formulation was deliberately adopted as a simple behavioral rule.

Note also that, in the organizational interpretation of the model, the payoff obtained in each interaction represents a local work-related benefit, such as productivity gains, recognition, access to opportunities, or other rewards generated by cooperative or competitive behavior. Consequently, the accumulated wealth $w_{i}\left(t\right)$ should be interpreted as a stylized measure of cumulative career advantage rather than as monetary income.

In the proposed model, reputation is assumed to decrease following defections and not to recover thereafter. This assumption was adopted to represent organizational contexts in which trust, once compromised, is difficult to rebuild within the time scale considered. Although reputation recovery may occur in real organizations through sustained cooperative behavior, incorporating such a mechanism would require the introduction of additional modeling assumptions, such as defining how many cooperative actions are needed to compensate for a previous defection and specifying the rate at which reputation is restored.

In short, type-1 agents are fixed probabilistic cooperators. In an organization, type-1 employees adopt a fixed probability of cooperation, regardless of past interactions, accumulated outcomes, or the behavior of others. Their actions are not strategically adjusted in response to experience or changes in the environment. Type-2 agents are adaptive probabilistic cooperators. In an organization, type-2 employees vary their probability of cooperation, tending to be more cooperative when their reputation declines and less cooperative as they accumulate more benefits or career gains. Their actions are strategically adjusted in response to their performance and progress toward their goal. In the model, the removal of an agent represents the dismissal of an employee from the organization.

## 3. Metrics

Population composition and wealth distribution are evaluated here using Shannon entropy [47,55] and the Gini coefficient [56,57].

Let $N_{1}\left(t\right)$ and $N_{2}\left(t\right)$ denote the number of type-1 and type-2 agents at time step *t*, respectively, with $N_{1}\left(t\right)+N_{2}\left(t\right)=N$. Define $n_{1}\left(t\right)=N_{1}\left(t\right)/N$ and $n_{2}\left(t\right)=N_{2}\left(t\right)/N$ as the corresponding fractions (evidently, $n_{1}\left(t\right)+n_{2}\left(t\right)=1$). The population composition at time *t* can be quantified by the normalized Shannon entropy $H_{n}\left(t\right)$, defined as:

(2) $$H_{n}\left(t\right)=\frac{-[n_{1}\left(t\right)\log n_{1}\left(t\right)+n_{2}\left(t\right)\log n_{2}\left(t\right)]}{\log2}$$

The extreme values of $H_{n}$ have the following interpretation: $H_{n}=1$ indicates an equal composition (50% of each type); $H_{n}=0$ corresponds to the extinction of one of the two types.

Let $W_{1}\left(t\right)$ and $W_{2}\left(t\right)$ denote the total wealth accumulated by type-1 and type-2 agents at time step *t*, respectively; i.e., $W_{1}\left(t\right)=\sum_{i=1}^{N_{1}\left(t\right)}w_{i}\left(t\right)$ and $W_{2}\left(t\right)=\sum_{i=1}^{N_{2}\left(t\right)}w_{i}\left(t\right)$, where the index *i* denotes the *i*-th agent of the corresponding type. Define $v_{1}\left(t\right)=W_{1}\left(t\right)/\left(W_{1}\left(t\right)+W_{2}\left(t\right)\right)$ and $v_{2}\left(t\right)=W_{2}\left(t\right)/\left(W_{1}\left(t\right)+W_{2}\left(t\right)\right)$ as the fractions of total wealth held by each type (thus, $v_{1}\left(t\right)+v_{2}\left(t\right)=1$). The wealth distribution between agent types can be quantified by the normalized Shannon entropy $H_{v}\left(t\right)$, defined as:

(3) $$H_{v}\left(t\right)=\frac{-[v_{1}\left(t\right)\log v_{1}\left(t\right)+v_{2}\left(t\right)\log v_{2}\left(t\right)]}{\log2}$$

Notice that $H_{v}=1$ reflects an equal distribution of wealth between the two types and $H_{v}=0$ implies complete concentration of wealth in a single type.

The average wealth per agent within the *i*-th type, $\alpha_{i}\left(t\right)$, is defined as the ratio of the total wealth to the size of the corresponding type. Thus, for type-1 and type-2:

(4) $$\begin{array}{ccc} \alpha_{1}\left(t\right) & = & \frac{W_{1}\left(t\right)}{N_{1}\left(t\right)} \end{array}$$

(5) $$\begin{array}{ccc} \alpha_{2}\left(t\right) & = & \frac{W_{2}\left(t\right)}{N_{2}\left(t\right)} \end{array}$$

Similar metrics have been employed in a previous study [44].

In addition, the Gini coefficient G(t) is computed to quantify the degree of inequality in the distribution of wealth for the entire population. This index ranges from 0, corresponding to an equal distribution of wealth, to 1, corresponding to complete concentration of wealth in a single agent. Here, it is computed as:

(6) $$G\left(t\right)=\frac{\sum_{i=1}^{N}\sum_{j=1}^{N}\left|w_{i}\left(t\right)-w_{j}\left(t\right)\right|}{2N\sum_{i=1}^{N}w_{i}\left(t\right)}$$

In the next section, results from numerical simulations of the proposed model are presented. The notation 〈x〉 used below denotes the average value of x(t) evaluated over the last $\tau_{1}$ time steps of a simulation with τ time steps. The focus of the present study is therefore on the long-term behavior of the system; hence, transient dynamical phases were not examined.

## 4. Results

Numerical simulations were performed on a 200×200 lattice (thus, *N* = 40,000), with initial conditions $n_{1}\left(0\right)=n_{2}\left(0\right)=0.5$ (i.e., the population initially consists of the two types in equal proportions). The payoff values of the prisoner’s dilemma are the canonical ones [1,5], T=5, R=3, P=1, and S=0. The agents were randomly distributed over the lattice at t=0.

**Figure 1:**
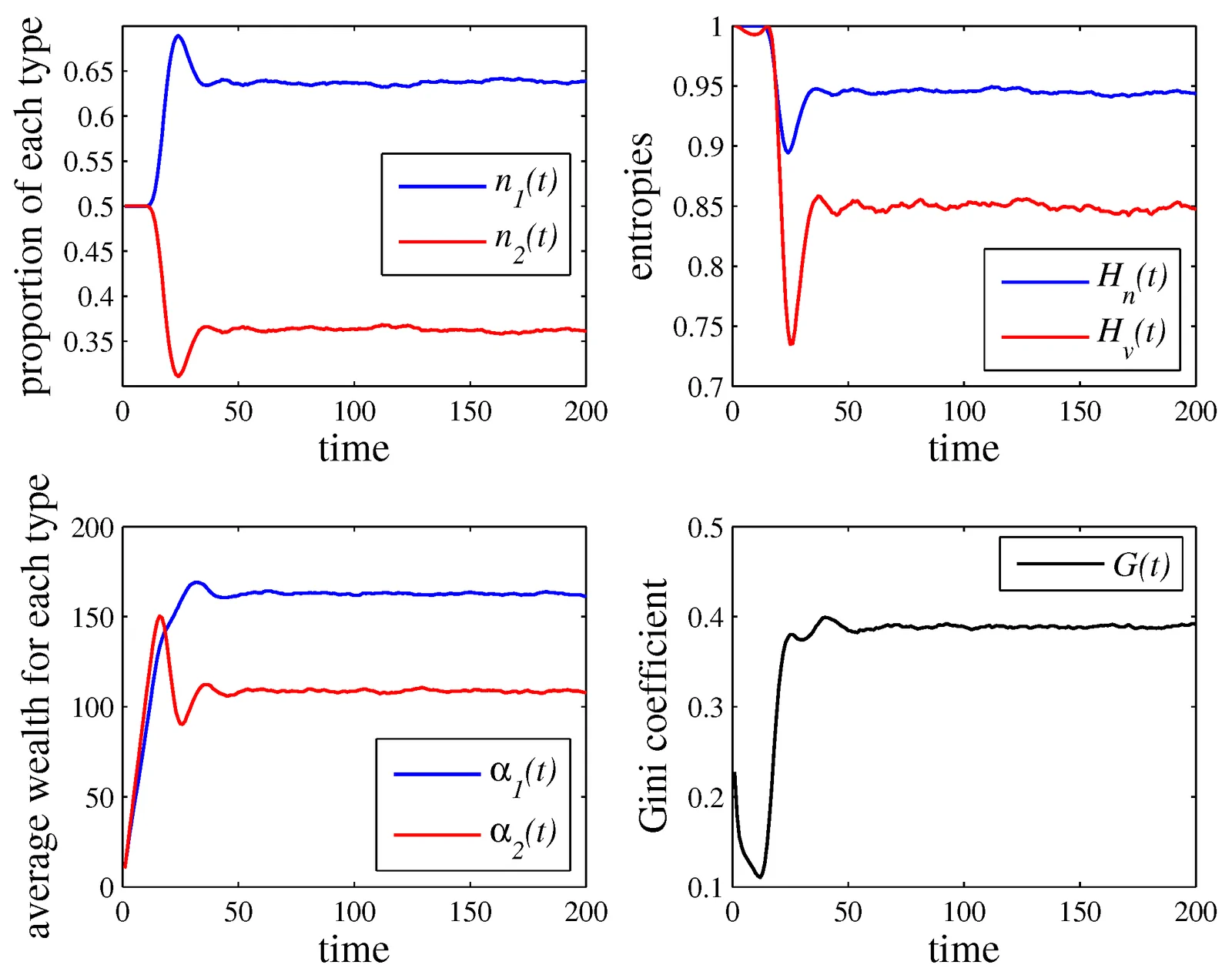
Time evolution of $n_{1}\left(t\right)$, $n_{2}\left(t\right)$, $H_{n}\left(t\right)$, $H_{v}\left(t\right)$, $\alpha_{1}\left(t\right)$, $\alpha_{2}\left(t\right)$, and G(t) for 0<t≤τ from the initial condition $(n_{1}\left(0\right),n_{2}\left(0\right))=\left(0.5,0.5\right)$. These plots were obtained with *N* = 40,000, T=5, R=3, P=1, S=0, q=0.7, g=50, $r_{0}=10$, and τ=200. In this simulation, type-1 agents tend to be both more prevalent and, on average, wealthier than type-2 agents.

Figure 1 illustrates the time evolution of $n_{1}\left(t\right)$, $n_{2}\left(t\right)$, $H_{n}\left(t\right)$, $H_{v}\left(t\right)$, $\alpha_{1}\left(t\right)$, $\alpha_{2}\left(t\right)$, and G(t) for 0<t≤τ. In this simulation, q=0.7, g=50, $r_{0}=10$, and τ=200. As time progresses, these variables fluctuate around the following average values: $\langle n_{1}\rangle =0.6375\pm 0.0009$, $\langle n_{2}\rangle =0.3625\pm 0.0009$, $\langle H_{n}\rangle =0.9450\pm 0.0007$, $\langle H_{v}\rangle =0.849\pm 0.003$, $\langle \alpha_{1}\rangle =162.9\pm 0.7$, $\langle \alpha_{2}\rangle =108.7\pm 0.6$, and 〈G〉=0.390±0.002. These average values were computed with $\tau_{1}=20$ (i.e., considering the last 20 time steps of the simulation). Note that, in this simulation, type-1 agents are more prevalent than type-2 agents (i.e., $\langle n_{1}\rangle >\langle n_{2}\rangle$), the population composition is more homogeneous than the wealth distribution between the two types (i.e., $\langle H_{n}\rangle >\langle H_{v}\rangle$), type-1 agents are on average wealthier than type-2 agents (i.e., $\langle \alpha_{1}\rangle >\langle \alpha_{2}\rangle$), and the Gini coefficient is approximately 0.4, indicating a moderate level of inequality in the wealth distribution across the population.

**Figure 2:**
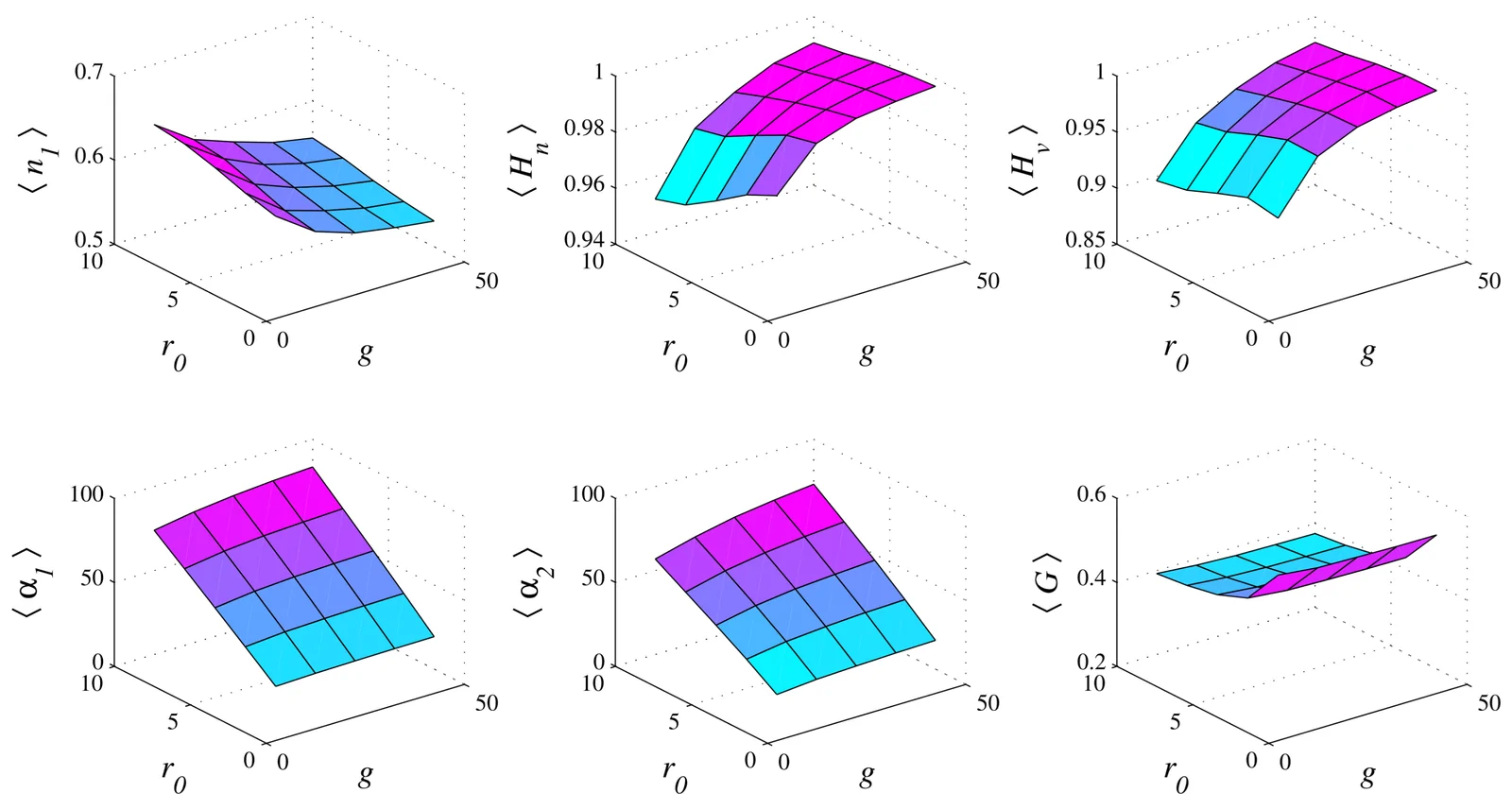
Long-term average values of $n_{1}$, $H_{n}$, $H_{v}$, $\alpha_{1}$, $\alpha_{2}$, and *G* computed for q=0.5, g={10,20,30,40,50}, and $r_{0}=\left\{2,4,6,8,10\right\}$.

**Figure 3:**
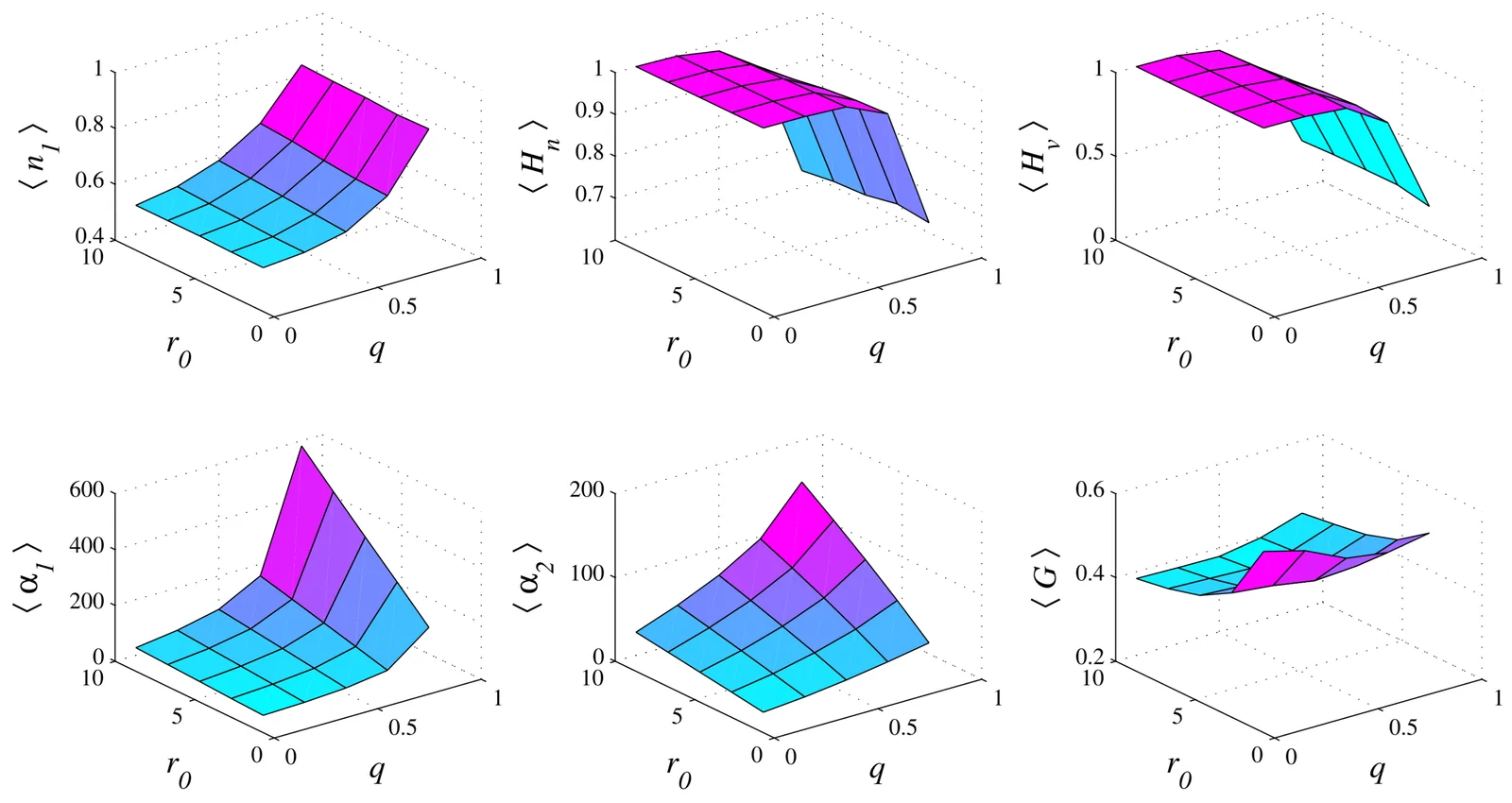
Long-term average values of $n_{1}$, $H_{n}$, $H_{v}$, $\alpha_{1}$, $\alpha_{2}$, and *G* computed for g=30, q={0.1,0.3,0.5,0.7,0.9}, and $r_{0}=\left\{2,4,6,8,10\right\}$.

**Figure 4:**
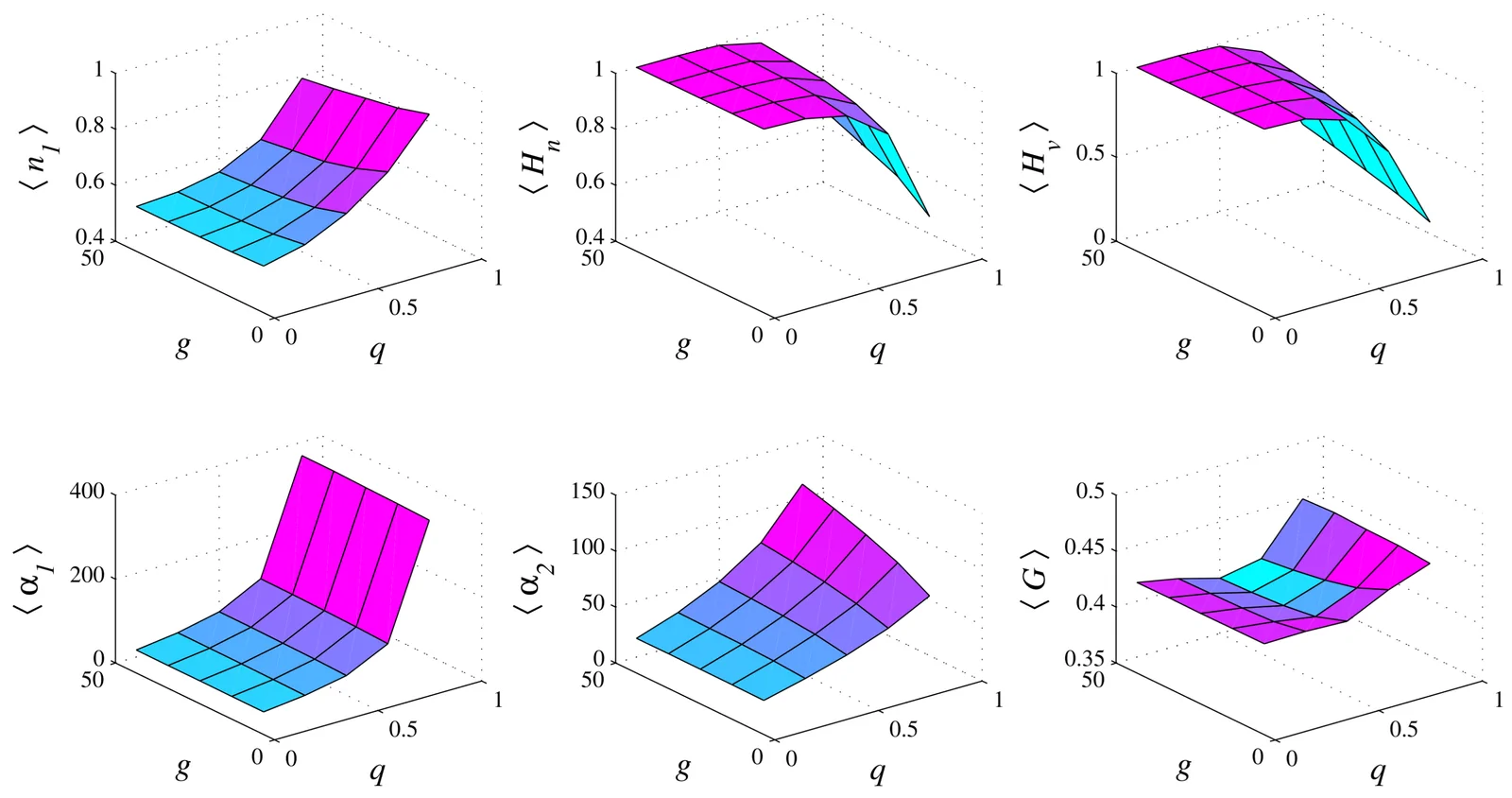
Long-term average values of $n_{1}$, $H_{n}$, $H_{v}$, $\alpha_{1}$, $\alpha_{2}$, and *G* computed for $r_{0}=6$, g={10,20,30,40,50}, and q={0.1,0.3,0.5,0.7,0.9}.

Figure 2, Figure 3 and Figure 4 present the influence of the parameters *q*, *g*, and $r_{0}$ on the long-term average values of the metrics defined in Section 3. Recall that *g* is the individual wealth goal to be reached by a type-2 agent, $r_{0}$ is the maximum number of defections that an agent of either type can make before being removed, *q* is the cooperation probability of type-1 agents, and higher values of *q* imply a higher cooperation probability for type-2 agents. The simulations corresponding to these figures were performed with τ=4000 and $\tau_{1}=50$ (thus, the averages were computed over the last 50 time steps of simulations with 4000 steps). For each set of parameter values, 25 independent simulations were performed and the average values were plotted in these figures. In Figure 2, q=0.5, g={10,20,30,40,50}, and $r_{0}=\left\{2,4,6,8,10\right\}$. In Figure 3, g=30, q={0.1,0.3,0.5,0.7,0.9}, and $r_{0}=\left\{2,4,6,8,10\right\}$. In Figure 4, $r_{0}=6$, g={10,20,30,40,50}, and q={0.1,0.3,0.5,0.7,0.9}. The standard deviation of the average values shown in these figures is typically below 0.5% of the corresponding mean. With 25 independent simulations, this implies a 95% confidence interval with a half-width of approximately 0.2% of the mean, indicating a high degree of consistency among independent simulations.

Figure 2 and Figure 4 show that increasing *g* decreases $\langle n_{1}\rangle$ (and thus increases $\langle n_{2}\rangle$), and also increases both $\langle H_{n}\rangle$ and $\langle H_{v}\rangle$. These changes indicate that the population composition and the wealth distribution between the two types become more balanced. However, *g* has little effect on $\langle \alpha_{1}\rangle$, $\langle \alpha_{2}\rangle$, and 〈G〉. Thus, in these simulations, *g* does not significantly affect the average wealth accumulated by agents of either type nor the overall level of inequality.

Figure 2 and Figure 3 show that increasing $r_{0}$ leads to increases in $\langle n_{1}\rangle$, $\langle \alpha_{1}\rangle$, and $\langle \alpha_{2}\rangle$, and to a decrease in 〈G〉. These results indicate that larger values of $r_{0}$ are associated with a higher prevalence of type-1 agents, higher average accumulated wealth for both types, and a reduction in wealth inequality across the population.

Figure 3 and Figure 4 also show that increasing *q* raises $\langle n_{1}\rangle$, leading to decreases in both $\langle H_{n}\rangle$ and $\langle H_{v}\rangle$. Therefore, larger values of *q* make both the population composition and the wealth distribution between the two types less balanced. Moreover, increasing *q* results in higher values of $\langle \alpha_{1}\rangle$ and $\langle \alpha_{2}\rangle$, indicating that agents of both types accumulate more wealth on average. The effect on the Gini coefficient, however, is non-monotonic, as shown in Figure 4.

Some quantitative results obtained from the simulations are:

- For q=0.5 and $r_{0}=6$, increasing *g* from 10 to 50 decreases $\langle n_{1}\rangle$ from 0.614 to 0.542, a reduction of approximately 11.7% (Figure 2).
- For q=0.5 and g=30, increasing $r_{0}$ from 2 to 10 decreases 〈G〉 from 0.523 to 0.380, corresponding to a reduction of approximately 27.3% (Figure 3).
- For g=30 and $r_{0}=6$, increasing *q* from 0.1 to 0.9 increases $\langle n_{1}\rangle$ from 0.504 to 0.831, an increase of approximately 65% (Figure 4).
- Under the same conditions, $\langle \alpha_{1}\rangle$ increases from 16.78 to 364.86 and $\langle \alpha_{2}\rangle$ from 16.74 to 94.96. Thus, the average wealth of type-1 and type-2 agents increases by factors of approximately 21.7 and 5.7, respectively (Figure 4).
- For g=30 and $r_{0}=6$, 〈G〉 changes from 0.417 at q=0.1 to 0.403 at q=0.5 and 0.449 at q=0.9, quantitatively illustrating its non-monotonic dependence on the cooperation probability (Figure 4).

**Figure 5:**
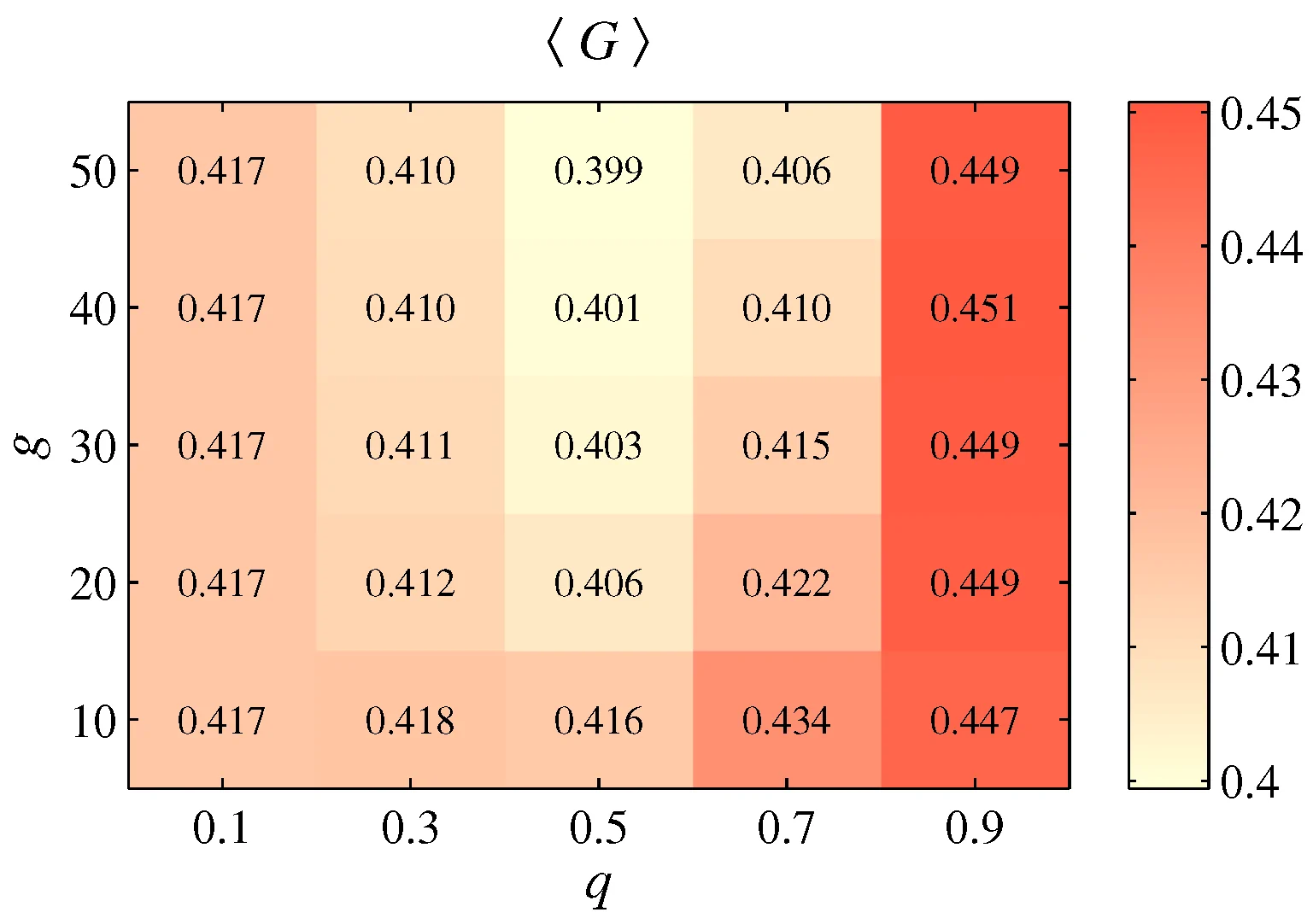
Heatmap of the average Gini coefficient 〈G〉 as a function of the cooperation probability *q* and the individual wealth goal *g*, for $r_{0}=6$. Each value represents the average over 25 independent simulations.

To provide a clearer visualization of the interaction between *q* and *g*, Figure 5 presents a heatmap of the average Gini coefficient over the (q,g) parameter plane. The heatmap confirms the non-monotonic dependence of 〈G〉 on *q* and shows that this dependence is also affected by *g*.

**Figure 6:**
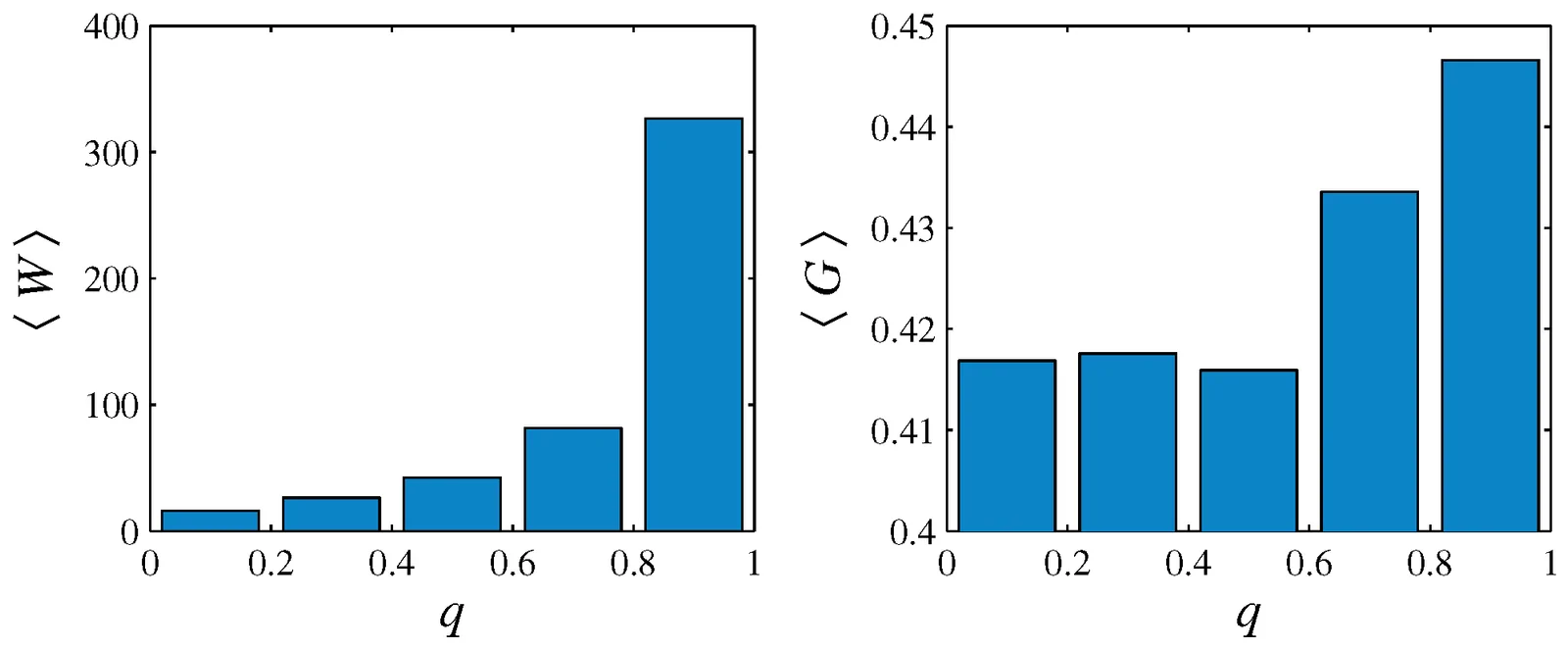
Long-term average values of *W* and *G* computed for $r_{0}=6$, g=10, and q={0.1,0.3,0.5,0.7,0.9}.

The Gini coefficient G(t) quantifies the degree of asymmetry in the wealth distribution within a population. However, a lower value of this index (i.e., a more uniform distribution) does not necessarily correspond to higher levels of available resources for individuals. Figure 6 presents the time-averaged values of G(t) and $W\left(t\right)=\alpha_{1}\left(t\right)n_{1}\left(t\right)+\alpha_{2}\left(t\right)n_{2}\left(t\right)$, where W(t) corresponds to the mean population wealth. In this figure, g=10, $r_{0}=6$, and q∈{0.1,0.3,0.5,0.7,0.9}. Note that increasing *q* leads to higher 〈W〉, but can also increase 〈G〉, despite small non-monotonic variations, indicating that greater cooperation does not necessarily reduce wealth inequality. Although wealth inequality is higher for q=0.9, the mean wealth is approximately 20 times that observed for q=0.1. In fact, the wealth of both types of agents increases with *q*; however, this effect is substantially more pronounced for type-1 agents, leading to a higher Gini coefficient.

## 5. Discussion and Conclusions

In the proposed model, there are two types of agents: fixed probabilistic cooperators, denoted as type-1, and adaptive probabilistic cooperators, denoted as type-2. Since the cooperation probability of type-2 agents is lower than (or equal to) that of type-1 agents, adaptive agents tend to choose cooperation less frequently, with the two types having the same cooperation probability when the accumulated wealth of type-2 agents is zero. From an organizational perspective, the model incorporates two opposing forces in the behavior of adaptive agents. For a given level of accumulated wealth, lower values of $r_{i}\left(t\right)$ increase the cooperation probability by reducing the term ${\left(r_{i}\left(t\right)/r_{0}\right)}^{2}$ in Equation (1). Conversely, for a given reputation level, larger values of $w_{i}\left(t\right)$ decrease the cooperation probability through the term $\left(w_{i}\left(t\right)/g\right)$. Type-2 agents represent a possible behavioral pattern in workplace environments, in which employees adjust their level of cooperation in response to both performance outcomes and reputational considerations.

The findings discussed below should be interpreted within the parameter ranges explored in the present study. The simulations indicated gradual changes in the long-term averages instead of sharp threshold-like transitions. The simulation results summarized in Figure 2, Figure 3 and Figure 4 show that type-1 agents are at least as prevalent as type-2 agents (i.e., $\langle n_{1}\rangle \geq \langle n_{2}\rangle$) and have at least as much accumulated wealth as type-2 agents (i.e., $\langle \alpha_{1}\rangle \geq \langle \alpha_{2}\rangle$). Figure 1 illustrates these typical results. This prevalence of type-1 agents suggests that maintaining a stable propensity to cooperate may provide a long-term advantage over adaptive strategies in which cooperation decreases as individual gains accumulate. These results suggest that incentive structures that decouple rewards from sustained cooperative behavior may generate short-term gains at the expense of long-term interaction efficiency.

Increasing *g* raises $\langle n_{2}\rangle$, making the population composition and the wealth distribution between types more balanced, with only minor effects on the average wealth accumulated by agents of either type. Increasing $r_{0}$ raises $\langle n_{1}\rangle$, $\langle \alpha_{1}\rangle$, and $\langle \alpha_{2}\rangle$.

The average Gini coefficient 〈G〉 decreases as $r_{0}$ increases, as shown in Figure 2 and Figure 3, because of the reduction in replacement events. Since newly introduced agents start with zero accumulated wealth, frequent dismissals continually reintroduce zero-wealth individuals into the population, thus increasing wealth disparities across the population. Larger values of $r_{0}$ allow agents to remain active for longer periods, providing broader opportunities for wealth accumulation and leading to a more homogeneous distribution of resources across the population. The weak dependence of the Gini coefficient on *g*, as shown in Figure 2 and Figure 4, suggests that individual wealth targets have a limited impact on the overall pattern of wealth inequality. Although *g* affects the persistence of type-2 agents, this influence does not seem to translate into major changes in the resulting wealth distribution.

The non-monotonic dependence of the Gini coefficient on the cooperation probability *q*, illustrated in Figure 4 and Figure 5, may reflect the interplay between two distinct mechanisms operating in different parameter regimes. For low values of *q*, frequent defections accelerate reputation loss, leading to higher rates of agent replacement. Since replacement resets accumulated wealth to zero, frequent dismissals increase the proportion of zero-wealth agents in the population, contributing to greater wealth disparities. As *q* increases to intermediate values, cooperation becomes more frequent, reputational losses occur less rapidly, and replacement events become less common. Under these conditions, wealth accumulation tends to be more homogeneous, resulting in lower values of the Gini coefficient. For high values of *q*, however, inequality increases again because type-1 agents, whose cooperation probability remains fixed, benefit more consistently from cooperative interactions than type-2 agents, whose cooperation probability decreases as their accumulated wealth approaches the individual wealth goal. Consequently, the observed U-shaped relationship between 〈G〉 and *q* suggests that distinct sources of inequality may dominate at low and high levels of cooperation. In this context, it should be emphasized that a higher cooperation probability may improve aggregate performance without necessarily promoting equity. In fact, a lower Gini coefficient does not necessarily imply higher average wealth among agents, as shown in Figure 6. These results stress that organizational efficiency and distributive equality need not vary in the same direction, and that incentive policies should be evaluated jointly in terms of productivity, persistence of cooperation, and the distribution of gains.

In organizational terms, the model suggests that employees who maintain a consistent propensity to cooperate tend to generate more favorable long-term outcomes than those whose cooperation decreases as individual gains accumulate. This result should not be interpreted as evidence that adaptive or goal-oriented behavior is intrinsically detrimental to organizational performance. Instead, it highlights a structural conflict between individual performance incentives and sustained cooperation. When performance targets are designed in a way that weakens cooperative behavior after rewards are achieved, they may undermine collective outcomes even as aggregate performance increases. The model therefore suggests that incentive systems focusing exclusively on individual accumulation may be less effective in sustaining long-term cooperation than systems that also include reputational or institutional feedback. In this sense, the coexistence of different behavioral profiles may remain compatible with favorable organizational outcomes when goals, rewards, and reputational dynamics are aligned with collective objectives.

This study can be further extended. As a preliminary investigation, simulations were performed in which dismissed agents were replaced by agents of the most prevalent type in their local neighborhood (from an organizational perspective, this implies that agents influence the profile of new hires, favoring candidates whose characteristics resemble those of the local majority). In this case, type-1 agents were always the most prevalent, and type-2 agents were eliminated in 56 of the 75 parameter combinations considered in Figure 2, Figure 3 and Figure 4. In another preliminary investigation, an alternative behavioral rule of type-2 agents was examined. In this case, when a type-2 agent reaches $w_{i}\left(t\right)\geq g$, it starts cooperating with the constant probability *q*, as a type-1 agent (from an organizational perspective, this represents employees who change their behavior after achieving their individual goal, becoming more consistently cooperative). The 75 parameter combinations considered in Figure 2, Figure 3 and Figure 4 were simulated using this alternative rule. Type-1 agents were more prevalent than type-2 agents in all cases, although type-2 agents were not eliminated in any of them. These preliminary results provide additional evidence that the predominance of fixed probabilistic cooperators persists under different modeling assumptions concerning employee behavior and replacement.

Further investigations may consider complex interaction networks, reputation recovery, smoother changes in cooperation probability as the individual wealth goal is reached, and greater heterogeneity in agents’ behavioral characteristics. A systematic sensitivity analysis of alternative functional forms for the cooperation probability, including additive dependence on wealth and reputation and different exponents for the reputation term, could also help determine the extent to which the results depend on the specific functional form adopted in Equation (1). Such extensions may help assess the robustness of the results under more general modeling assumptions and broaden the applicability of the model to different organizational settings.

## Data Availability

The data that support the findings of this study are available upon reasonable request.
